# Challenges of disseminating clinical practice guidelines in a weak health system: the case of HIV and infant feeding recommendations in Tanzania

**DOI:** 10.1186/s13006-014-0024-3

**Published:** 2014-12-23

**Authors:** Elizabeth H Shayo, Bodil Bø Våga, Karen Marie Moland, Peter Kamuzora, Astrid Blystad

**Affiliations:** Department of Global Public Health and Primary Care, University of Bergen, PO Box 7804, 5020 Bergen, Norway; National Institute for Medical Research, PO Box 9653, Dar-es-Salaam, Tanzania; Department of Health Studies, University of Stavanger, 4036 Stavanger, Norway; Institute of Development Studies, University of Dar-es-Salaam, PO Box 35169, Dar-es- Salaam, Tanzania

**Keywords:** Clinical guidelines, Communication challenges, Healthcare system, The PMTCT programme, Tanzania

## Abstract

**Background:**

Clinical guidelines aim to improve patient outcomes by providing recommendations on appropriate healthcare for specific clinical conditions. Scientific evidence produced over time leads to change in clinical guidelines, and a serious challenge may emerge in the process of communicating the changes to healthcare practitioners and getting new practices adopted. There is very little information on the major barriers to implementing clinical guidelines in low-income settings. Looking at how continual updates to clinical guidelines within a particular health intervention are communicated may shed light on the processes at work. The aim of this paper is to explore how the content of a series of diverging infant feeding guidelines have been communicated to managers in the Prevention of Mother to Child Transmission of HIV Programme (PMTCT) with the aim of generating knowledge about both barriers and facilitating factors in the dissemination of new and updated knowledge in clinical guidelines in the context of weak healthcare systems.

**Methods:**

A total of 22 in-depth interviews and two focus group discussions were conducted in 2011. All informants were linked to the PMTCT programme in Tanzania. The informants included managers at regional and district levels and health workers at health facility level.

**Results:**

The informants demonstrated partial and incomplete knowledge about the recommendations. There was lack of scientific reasoning behind various infant feeding recommendations. The greatest challenges to the successful communication of the infant feeding guidelines were related to slowness of communication, inaccessible jargon-ridden English language in the manuals, lack of summaries, lack of supportive supervision to make the guidelines comprehensible, and the absence of a reading culture.

**Conclusion:**

The study encountered substantial gaps in knowledge about the diverse HIV and infant feeding policies. These gaps were partly related to the challenges of communicating the clinical guidelines. There is a need for caution in assuming that important changes in guidelines for clinical practice can easily be translated to and implemented in local programme settings, not least in the context of weak healthcare systems.

## Background

The drive towards ‘evidence-based practice’ has increased the demand for standard procedures in healthcare provision globally. Global and national clinical guidelines are being developed and revised at an increasing speed as new evidence emerges. In many clinical areas knowledge is short-lived, and new evidence calls for the frequent revision and updating of clinical guidelines. HIV and infant feeding has been a particularly dynamic and demanding area of research, and, between 1992 and 2013, new evidence has regularly been incorporated into global and national guidelines leading to a fast pace of change. In this paper, we discuss the challenges related to the dissemination of frequently changing guidelines in weak healthcare systems. Specifically, we explore infant feeding guidelines and their dissemination and adoption in the Prevention of Mother-to-Child Transmission of HIV (PMTCT) Programme in Tanzania as a case study.

The aim of clinical guidelines is to improve the quality of care and health outcomes for patients by translating new research findings into practice [1,2]. The guidelines are to assist practitioners and patients in making appropriate healthcare decisions for specific circumstances [3]. They should be based on the ‘best evidence’ available and should be implemented with clinical expertise combined with fundamental consideration of patients’ preferences.

Research continually produces new findings that may contribute to more effective and efficient healthcare. However, this new knowledge cannot change patient outcomes unless health services and health workers adopt the findings in practice [4]. Translating evidence into practice, i.e. the implementation of new policy and knowledge, is an active process involving individuals, teams and organizations [5]. Such processes can be challenging since they involve changes in knowledge, attitudes and behaviour. In this context, identifying barriers is an important step in the process of knowledge translation [6]. Although governments, professional associations and healthcare organisations commonly play an active part in the development and dissemination of clinical guidelines [7], there is often a substantial gap between procedures detailed in guidelines and actual healthcare practice [8]. In their review, Grol and Grimshaw [8] pointed out the different levels in the healthcare systems where challenges may occur; at the level of the patient, the healthcare professional, the healthcare team, the healthcare organisation, and within the wider environment. In the implementation of evidence/knowledge in healthcare it is important to understand the obstacles in order to develop effective channels of communication/information and, in turn, improve health interventions.

A number of concrete barriers have been identified which hinder the smooth adoption of clinical guidelines. These include lack of awareness of the existence of the guidelines among care providers, a lack of agreement between providers and experts about the scientific evidence behind the changes, and a lack of ability to adopt new medical practices into daily routines [9-11]. In a systematic review that involved studies outside Africa, almost all studies revealed that the quality of care did not attain the standard set in national guidelines or those set by the researchers themselves [12]. Jackson and Feder [3] write that characteristics of good guidelines include their ability to present evidence and recommendations in a concise, accessible manner and in a format which facilitates quick retrieval and assimilation of information. Guidelines should present relevant and valid evidence for the clinicians to relate to. They should be relevant to patients and they should be presented in a format that is flexible enough to be applicable to specific patients or circumstances.

Our findings will draw upon Jackson and Feder’s findings, and will be discussed in-depth by drawing upon the ‘diffusion of innovations theory’ developed by Rogers [13], a theory which enhances the understanding of the dissemination of policy. At a very general level, Rogers underscores the fundamental importance of considering the potential ‘benefits and costs’ of health ‘innovations’ before implementing them. The theory suggests five elements which should be carefully considered during the phase of assessing the feasibility of new health policy, namely the innovation’s (1) ‘relative advantage’, its (2) ‘compatibility’, (3) ‘complexity’, (4) ‘trialability’ and (5) ‘observability’ (see Table 1). Roger’s five elements will guide our discussion of the study findings.

**Table 1:** **Five elements to be considered in the diffusion of innovation theory**

**Elements**	**Details**
Relative advantage	The degree to which an innovation is perceived as better than the idea it supersedes.
Compatibility	The degree to which an innovation is perceived as being compatible with existing values, past experiences, and the needs of potential adopters.
Complexity:	The degree to which an innovation is perceived as difficult to understand and use. A clinical procedure is more likely to be adopted if it is simple and well defined.
Trialability	The degree to which the innovation may be piloted to explore the implementation of the procedure, its acceptability to patients, and the potential outcomes.
Observability	The degree to which the results of the innovation are visible to others.

Infant feeding guidelines in the PMTCT programme provide us with an interesting case for looking at the process of introducing globally defined guidelines (in this case produced by WHO) into constrained health systems. This case illuminates the particular challenges of frequent and radical changes pertaining to health policy implementation in the global south.

### Global infant feeding guidelines in PMTCT programmes

Acting upon the evidence that HIV can be transmitted through breastfeeding [14,15], the World Health Organization (WHO) developed a series of global infant feeding guidelines on how an HIV positive mother should feed her infant. The guidelines targeted policy-makers, health workers and HIV-infected mothers. Over time, these series of guidelines involved major changes in the infant feeding recommendations for women living with HIV.

WHO issued new infant feeding guidelines in 1992, 1997–98, 2000–01, 2003, 2006, 2009, 2010, 2012, and 2013. The major changes and the rationale underlying the changes can be summarized as follows:

- 1992: Breastfeeding was strongly encouraged for both HIV-infected and non-infected women with the rationale that the risk of malnutrition and death in infants from not breastfeeding outweighed the risk of HIV infection from breastfeeding [15].
- 1997: Choice of infant feeding method by mothers was encouraged after they had been counselled about the risk of breastfeeding and the risks of not breastfeeding. The rationale was based on new evidence about the risk of HIV transmission through breastfeeding, coupled with a stronger emphasis on patients’ rights to participate in healthcare decision-making [16].
- 2000–01: Exclusive replacement feeding (formula feeding) was recommended as the first option for HIV-infected mothers if found to be ‘acceptable’, ‘feasible’, ‘affordable’, ‘sustainable’ and ‘safe’ (AFASS) in the particular context [17] owing to fear of exposure to HIV in mothers’ milk. In cases where formula/replacement feeding was not considered to be AFASS, exclusive breastfeeding with rapid cessation at six months was recommended in order to reduce the risk of mixed feeding
- 2003: Exclusive breastfeeding for the first six months of life was recommended as the first infant feeding choice for HIV-infected mothers due to the improved HIV free survival with exclusive breastfeeding [17,18] including the recommendation of abrupt cessation of breastfeeding after six months to prevent mixed feeding patterns.
- 2006: An extended period of breastfeeding was allowed for. The rationale behind the change was an increasing number of studies that documented improved HIV-free survival with exclusive breastfeeding [18-22], combined with evidence of increased death and malnutrition in formula-fed infants.
- 2010: Twelve months of breastfeeding was recommended (six months exclusive breastfeeding and the introduction of complementary feeds thereafter) [23]. This change was based on evidence showing the efficacy of antiretroviral drugs (ARVs) in preventing postnatal transmission during breastfeeding [24].

Most countries have largely adopted the HIV and infant feeding guidelines developed by the WHO with only minor modifications. Tanzania is an important case in point. Table 2 briefly summarises the main WHO policy shifts, and Tanzania’s response to them.

**Table 2:** **Summary of infant feeding changes of the global infant feeding guidelines for HIV infected mothers**

**YEAR**	**World Health Organization**	**Tanzania**
1992	• Breastfeeding	• Adopted
1997	• Right to choose either to breastfeed or use replacement milk	• Adopted
2000/2001	• Replacement feeding first option when AFASS criteria are met	• Adopted
2003	• Exclusive breastfeeding for the first 6 months first option; abrupt cessation at 6 months	• Adapted in 2003: Animal milk was encouraged for replacement fed children
	• No mixed feeding; alternatively replacement feeding using formula or animal milk; heating of mother’s milk	• Heating of mothers’ milk not recommended
2006	• Exclusive breastfeeding for the first 6 months first option; extend breastfeeding if replacement feeding is not AFASS	• Adapted in 2007, heating of mother’s milk and wet nursing not recommended.
	• No mixed feeding for the first 6 months of age; alternatively replacement feeding using formula or animal milk; heating of mother’s milk; wet nursing	
2010	• Breastfeeding for 12 months: i.e. exclusively for the first six months then introduce complementary foods thereafter	• Adapted in 2011
	• ARVs are administered during the breastfeeding period; Express and heat mothers’ milk if ARVs not available; Gradual weaning	
	• Decision regarding feeding option is left to the country	
	• Animal milk strictly prohibited	

### The PMTCT programme in Tanzania

The PMTCT programme was introduced in Tanzania in 2000, starting with referral hospitals as pilot areas, followed by a rapid nation-wide scale-up from 2003 and onwards. The programme relies largely on donor support. The implementation of the programme has been guided by national guidelines that to a considerable extent have followed the changing WHO recommendations (as indicated in Table 2) [25-27]. The PMTCT services provided in Tanzania have aimed to include routine HIV testing and counselling, antiretroviral (ARV) treatment and prophylaxis for mothers and children, safer delivery practices, counselling and support for safer infant feeding practices, and long-term follow-up care for mother, child and family. The programme is implemented within health facilities that provide reproductive and child health (RCH) services including hospitals, health centres and dispensaries, both public and private. The policy has been to train at least one member of staff on PMTCT and its subsequent changes in every health facility providing PMTCT services.

In 2013 Tanzanian authorities reported, without any reference to quality or structure, that 96% of the RCH facilities had integrated the PMTCT programme, albeit with variations in implementation rates across the country [28]. During the decade of PMTCT programme implementation, Tanzania has seen a positive trend towards the reduction of HIV infection among pregnant women from 6.9% in 2008 to 3% in 2012 [29]. Despite the reduction in HIV prevalence, the country has experienced substantial challenges related to preventing post natal transmission, particularly related to breastfeeding. A number of studies have documented implementation challenges, especially of the 2001 guidelines, which, with their recommendation of early and abrupt weaning, were most radically different from customary feeding patterns of prolonged breastfeeding with early initiation of supplements [30,31]. Other than the studies mentioned, there is a dearth of information about the dissemination process during this period of rapidly and radically changing policies and clinical guidelines on HIV and infant feeding. Our study aimed to explore the ways in which changes in the infant feeding guidelines have been communicated to and understood by the regional and district level managers of the PMTCT programme as well as the health workers in charge of the PMTCT programme at the health facilities.

## Methods

### The study setting

The study was conducted in Mbarali District in Mbeya Region in 2011. According to the 2012 National Population Census, Mbarali has a population of 300,517 people (males =145,867, females = 154,650) [32] with a growth rate of 2.7%. The district is predominantly rural, and is populated largely by rice cultivating farmers from Sangu, Hehe, Bena and Nyakyusa ethnic groups and livestock keepers from Sukuma and Maasai ethnic groups. The district is served by both public and private health facilities: two hospitals (one public and one private), four health centres and 43 dispensaries. The coverage of the PMTCT programme services, introduced in the district in 2005, was 56% in 2008 [33], but was, during the study period, scaled up to 86%, i.e. to 32 of the 37 health facilities providing Reproductive and Child (RCH) health services (2011, personal communication by the first author).

Mbarali was chosen as a study site because the infant feeding sub-project was part of a larger EU- funded health systems research project, REACT - “Response to accountable priority setting for trust in health systems”. REACT assessed the application of the ethically based priority setting framework ‘accountability for reasonableness’ (AFR) [34]. HIV/AIDS was one of the focus areas of the project. The Mbeya region has a HIV prevalence of 9%, well above the national estimates of 5.1% [29]. The PMTCT programme seemed to provide a particularly interesting point of departure because of the many and substantial policy changes within infant feeding that had characterized this high-profile global health intervention.

#### Study design

Because the project aimed at generating knowledge about the dissemination and communication of information about clinical guidelines within the health system, a qualitative design with an exploratory approach was deemed appropriate. A triangulation design was chosen, combining individual in-depth interviews (IDIs) and focus group discussions (FGDs). The focus groups were used in an attempt to probe deeper into the findings in the IDIs and to enhance the validity of the findings.

#### Recruitment of informants

We aimed to interview individuals who were centrally located within the running of the PMTCT programme. All study participants had experience from different levels of administration of or more concrete management of the PMTCT programme. Regional, district and health facility levels were targeted as these are the administrative levels with most direct contact with the PMTCT programme. The main categories of informants included: 1) members of the regional management team (three individuals); 2) members of the district management health team, both permanent and co-opted members (nine individuals); and 3) PMTCT in-charges at health facilities (10 individuals, five in rural and five in semi-urban settings). In addition, two FGD focus group discussions were carried out with health workers at the health-facility level (public and faith-based hospitals) with 12 and 10 participants respectively (Table 3). The aim of the FGDs was to discuss the findings from the IDIs to increase the knowledge gained from the health workers who have the day to day contact with the women targeted by the policies. All the regional informants and six from the district level had followed the PMTCT programme since its introduction in their respective areas. However, at the health facilities variations existed depending on the period when the programme was introduced, since the scaling-up started with hospitals, then health centers followed, and lastly the dispensaries with RHC services. Three district officials and two health workers had experience of less than two years. All informants could recall some of the changes that had taken place in the infant feeding guidelines, and a majority of them received some training on PMTCT management.

**Table 3:** **Data collection methods and number of informants**

**Data collection method**	**Informants**	**No. of IDIs/FGDs**	**No. of participants**
In-depth interviews (IDIs)	Health facility PMTCT in-charges	10	10
	District managers	9	9
	Regional managers	3	3
Focus Group discussions (FGDs)	Health workers from faith-based institution	1	10
	Health workers from government institution	1	12
**Total participants**			**44**

The District HIV/AIDS focal person helped to identify and select the health facilities and potential care providers we could approach for participation in the study. The District Medical Officer facilitated the identification of potential district and regional informants. We recruited informants at health facility level from both rural and urban areas. The exact number of informants was not pre-determined; recruitment continued until there was a general sense that major themes/findings were repeated, indicating a general level of data saturation. The topic did not emerge as particularly sensitive, and the recruited informants were highly engaged in the study topic. No one who was approached declined to take part in the study.

#### Data collection

An interview guide was developed for the IDI’s (with some variation pertaining to level), and a broader topic guide was developed for the FGDs. The guides focused on the following main themes: *(1) awareness about the changes and content of the various infant feeding policies for women living with HIV, (2) knowledge about the reasons behind the various policy changes (3) information provided to relevant stakeholders about the HIV and infant feeding policy at various points in time and (4) reflections on the way the HIV and infant feeding policies and guidelines were communicated.*

While it is difficult to avoid bias in qualitative studies, an effort was made to reduce it. The interviews were conducted in venues within the office premises of the interviewees but in a separate room in attempts to enhance the freedom of expression, confidentiality, privacy and noise reduction. The study participants in both the IDIs and the FGDs eagerly engaged in the discussion about the information received about the PMTCT guidelines, and the large majority had lots of experience and opinions to share. Each IDI and FGD lasted between one and two hours. Emphasis was placed on letting the participants discuss without interruption, allowing for emerging responses and probing. In the FGD an effort was made to ensure that no single participant dominated the discussion. At the closing of each theme the moderator (the first author) made attempts to sum up the main points brought up, asking for the participants’ feedback.

To enhance validity and further reduce potential bias of the informants’ responses, all interviews/discussions except three IDIs were tape recorded with the permission of the informants.

#### Data analysis

In qualitative research, the data analysis starts in the field, implying continuous interpretive processes. The IDIs and FGDs were transcribed verbatim by a competent social scientist with previous experience of transcription. The first author carefully read all the transcripts and listened to the audio-tapes to confirm what was written and to gain a sense of the main emerging themes. The second stage involved close re-reading of each transcript, developing codes by summarizing the content of each sentence or sequence of text seeking to capture the key idea in each section. To as large an extent as possible the informants’ own expressions were employed as codes. The last author of this paper also read a number of the transcripts, and the codes developed were carefully discussed between the first and the last author to enhance coherence. During the next stage, the codes were sorted into larger categories, and were finally linked to the main themes brought up in the interview guides such as ‘awareness of the changes in the infant feeding guidelines’, ‘the reasons behind the changes’; and ‘communication challenges’. The data analysis was carried out manually. Detailed matrices containing the key findings were created, making it possible to detect evolving responses and similarities and differences among and between the different levels of informants. Triangulation in data collection aims to increase the understanding of complex phenomena using different sources [35]. In this study triangulation of data from the IDIs and FGDs was carried out in a manner where information gained in the IDIs was dwelled upon in the FGDs in attempts at refining, nuancing and validating the findings.

#### Ethical considerations

The study obtained ethical clearance from the Medical Research Co-ordinating Committee (MRCC) of the National Institute for Medical Research in Tanzania (NIMR/HQ/R.8a/Vol. IX/1094). Permission to conduct the study was also received from the regional and district authorities in Mbeya and Mbarali. Oral informed consent was sought from informants at all levels after the aims of the study had been explained. Informants were assured of the voluntary nature of participation, and of their right to withdraw from the discussion at any time without consequences. The principles of privacy and confidentiality were strictly maintained throughout the study.

## Results

### Misconceptions and lack of knowledge about the PMTCT guidelines

The findings section emphasizes the prime study findings; namely the many challenges experienced in receiving sufficient information about the continuously changing HIV and infant feeding guidelines. The regional informants were aware of the entire period of HIV and infant feeding guidelines, including the relatively recent 2010 guidelines, so the interviews with this category covered the entire period the PMTCT programme had operated in Tanzania. With the district and health facility informants, the period from 2005 was discussed as this was when the PMTCT programme was introduced in Mbarali. Before presenting the challenges of communication experienced, we will briefly discuss the understanding of the infant feeding policies that we encountered among the study participants.

Despite there being an understanding of the major policy changes in the PMTCT infant feeding recommendations during the past decade, we found serious gaps of knowledge at every administrative level included in the study. Knowledge gaps, for example, included the serious misunderstanding that the 2000 WHO guidelines promoted replacement feeding only, i.e., the informants were not aware that the policy at this time presented an option of breastfeeding if replacement feeding was not considered to be feasible. This misunderstanding was found as high as at the regional level. The changes that were introduced in the 2003–2007 policy were thus perceived as a move from an ‘*authoritative*’ (providing no choice) to a more ‘*friendly*’ approach providing HIV-infected mothers with alternatives – alternatives that had, in reality, been in the PMTCT guidelines all along.

A fundamental lack of awareness of the scientific explanations behind the many policies was a theme that ran throughout the material. For example, the scientific basis behind abrupt vs. gradual weaning, or behind the recommendations of first allowing for and later removing the possibility of using animal milk as replacement product. Serious misunderstandings were moreover encountered: “*Research evidence has revealed that abrupt cessation or early cessation increases the risks of HIV transmission to the babies*” (IDI-regional informant).

Vital policy changes were not widely known among the informants. For example, few informants knew that the 2007 Tanzania guidelines opened up for HIV-infected mothers to continue breastfeeding after six months, and to introduce complementary foods until they could wean their infants safely. When asked about the most recent recommendations, most of the informants answered six months of exclusive breastfeeding with abrupt cessation, a policy that had long since been replaced in Tanzania. Importantly however, all the study participants expressed a strong belief in exclusive breastfeeding being linked to the observation of HIV free babies born by HIV infected mothers.

### Top down communication challenges

Informants were asked about the ways in which changes on infant feeding guidelines had been or were presently communicated. At the regional level, stakeholders were informed by national managers through workshops and meetings, whereas at the district and health facility levels healthcare providers were informed by regional managers through training. Training sessions had, for example, been provided on testing and counselling, on how to administer ARVs as well as on how to advise mothers on best feeding practices, how to prepare replacement milk and on how to ensure early infant diagnosis. In both IDIs and FGDs at health facility level, this training was said to be characterized with a top-down approach, and care providers did not feel they were given sufficient opportunity to question or comment upon the recommendations.

The challenges encountered in communicating the continual modifications were increased by confusion over how to deliver messages that in themselves were difficult to grasp.

#### Communicating a complex public health message: “. . . there is a likelihood that they will forget”

The enormous challenge of trying to communicate a public health message that was perceived to be not feasible in the local context ran through the discussion. The district and health facility informants particularly emphasised the difficulties in delivering the message about formula feeding to the HIV-infected mothers. Most of the mothers were from rural areas with limited education and very low incomes. Thus, the care providers in both FGDs and IDIs explained that it was difficult for these mothers to meet the standards required for replacement feeding:

“*The information about the preparation of replacement feeding is difficult for mothers to understand… This might be taken by policy-makers as a challenge, and alternative ways should be sought rather than (merely promoting) issues of heating mother’s milk*” (IDI-district informant).

After the substantial difficulties faced in delivering the messages on replacement feeding, regional informants found the 2011 recommendations that allowed for an extended breastfeeding period reassuring;

“*We cannot teach care providers to tell mothers to rely on replacement milk. It is difficult because the criteria required for replacement feeding are difficult for rural women to understand; there is a likelihood that they will forget when they are required to adhere to all of them*” (IDI-regional informant).

#### Communicating a constantly changing message: “. . . that is where the confusion started”

The frequent changes in the infant feeding guidelines were said to confuse the PMTCT managers. It was perceived to be challenging to deliver new messages in a comprehensible and trustworthy manner to lower level care providers, who in turn would have to present the information to the HIV-infected mothers. All managers at regional level complained about this issue.

“*In 2000, we were entrusted with advising HIV-infected mothers not to breastfeed as the risks of transmitting infection were high. Later on we received training, and that is where the confusion started, because we were asked to advise HIV-infected mothers to breastfeed rather than rely on supplementary milk*” (IDI*-*regional informant).

A regional informant was also frustrated about the changes, fearing for his reputation:

“*At one time you tell them ‘don’t allow mothers to breastfeed’, another time you come and insist strongly ’you should advise mothers to breastfeed’. Care providers might consider you confused*” (IDI- regional informant).

Another regional informant revealed similar frustration regarding the changing messages, this time relating to the acceptance of vs the banning of animal milk:

“*These changes have brought about confusion, because in previous training we taught health workers that animal milk is recommended as it is affordable by the majority of the women, in contrast to formula milk. Now the new guidelines have banned the use of animal milk, and we are supposed to train healthcare providers. . . We don’t know how we are going to make it clear*” (IDI-regional informant).

#### Lacking reasons behind the health message: “. . . I haven’t heard of any reasons behind that”

The tendency of not providing reasons or sufficient explanation for policy changes within the PMTCT programme was reported to be common in the communication surrounding the guidelines. The study informants expressed that being provided with the reasons behind the various policy shifts would have facilitated important clarifications during the training, and would have eased the education of the mothers:

“*Different guidelines are produced based on the research evidence. But we managers have never seen a person from the Ministry of Health telling us that the guidelines have changed because of 1, 2, 3, etc*”. (IDI-regional informant).

Another explained:

“*We have been told that animal milk is no longer recommended. I haven’t heard of any reasons behind that. . . We were just told that ‘from now on children below six months of age should not feed on animal milk. It was just a single sentence on the slide in the power point presentation*” (IDI-regional informant).

Lack of explanation of the reasons for changes was found to contribute to superficial and confusing health messages:

“*For example there are messages like. . . ‘You (speaking to a mother) just squeeze your breast milk, put it in a pan, and then heat it and all viruses will die’. Messages like this one will definitely confuse the mothers*” (IDI-district informant).

Such statements were seen to be far too shallow to allow for a proper comprehension of the particular behavioural change called for (although such message was not recommended in the Tanzanian guidelines).

A limited number of health workers in each health facility, usually only one of higher rank, was trained on the many and constantly changing guidelines. As a result, the lower cadres found it difficult to understand the rationale behind the changes as they were only briefly oriented onsite, causing mistakes of various kinds:

“*You know very few staff received training. . . For example there was an HIV infected woman who was to give birth when I was out of the office; I instructed the nurse assistant on duty to provide the medicines to the mother and the child. She didn’t do so because she didn’t understand why the mother should also get medicines*” (IDI, health facility informant).

#### Guidelines clouded by English academic jargon: “. . . they are more for academia”

Informants who had been exposed to one or more of the versions of the PMTCT guidelines found the language difficult to comprehend.

“*If you open the PMTCT guidelines [researcher saw it on the table], their page numbers and the way they are written… there is no way our care providers will understand them. These guidelines need a person who has gone to school up to an advanced level.*” (IDI-district informant).

District managers found it difficult to translate or simplify the guidelines as the English language is not used in daily communication in Tanzania:

“*It is extremely difficult to translate the guidelines from English into Kiswahili to meet healthcare providers’ needs at the health facility level*” (IDI district informant).

Another district officer explained:

“*Most of our facility staff has a lower level of education, so if you give them guidelines in an English version they just put them on the table without reading them. Unless we managers provide thorough supportive supervision to clarify some of the issues outlined in the guidelines facility, staff will not understand them*” (IDI-district informant).

Whereas the regional informants interviewed did not have a problem with the language used in the guidelines, informants at the district and health facility levels found the use of English language in the development of the PMTCT guidelines prohibitive for their understanding. Indeed, the majority of the health personnel at the health facilities, the first line implementers of the guidelines, expressed that they simply did not understand the content. District informants thus found it difficult to provide refresher training to health workers employing the guides:

“*Training facility staff requires that the trainers understand the guidelines thoroughly so that care providers can receive the messages correctly and consistently, (and that is not the case)*” (IDI-district informant)*.*

The care providers complained about the lack of abridged guideline versions. Posters with easy steps to take when HIV infected mothers turn up for delivery were said to be available at the hospital’s maternity section only. With no summaries to help them understand the core issues, many care providers felt that they were not in a position to pass on knowledge properly about the content of the changing PMTCT guidelines. In fact, informants at the district and facility levels had difficulty understanding how many of the changes presented in the guidelines reflected the realities in Tanzanian communities; “*The guidelines are more theoretically based; they are more for academia, and don’t reflect the realities in our communities*” (IDI-facility informant)*.* Moreover, some informants suggested that the PMTCT guidelines might reflect the interests of the donors supporting their ARV provision.

#### A missing ‘reading culture’: “. . . but have they opened the manuals and read them?”

Aggravating the challenge of manuals that were not readily accessible was a reported lack of a culture of reading, even among district and health facility staff. This was a point identified by regional informants as another major limitation in a communication context:

“*Some care providers think that to implement anything there must be training. For example the manual has explained the use of combination ARVs since 2007, but the health facility providers are still prescribing a single dose. If you ask them they respond… oh … I haven’t received training … but have they opened the manuals and read them?*” (IDI-regional informant).

The implication of this lack of using the manuals was a lack of understanding and the spread of rumours or hearsay like: “*mothers’ milk should be warmed for some minutes to kill the virus instead of feeding the baby directly from the breasts’*” (IDI, district informant). This kind of statement was commonly heard, despite the fact that the Tanzanian guidelines do not recommend the pre-heating of mothers’ milk.

#### Lacking administrative procedures: “Here are your books”

The lack of sufficient knowledge was partly linked to a perceived lack of supportive supervision from the district level. Most of the informants at district and health facility levels also reported the lack of a clear administrative structure that would facilitate a smooth flow of communication of information. This led to poor distribution of PMTCT related information to the lower levels:

“*You can receive a phone call from the region; if you go there they tell you ‘here are your books’, and if you open them you find that they are guidelines. In the district, I also circulate them to the health facilities without any discussion, because even those who bring them simply just dump them*” (IDI-district informant).

At the district level, the informants reported poor links between the departments that play direct and indirect roles in the PMTCT programme, making it difficult to enforce the implementation.

#### Missing clinical PMTCT guidelines: “We implementers have never seen it”

Some versions of the PMTCT guidelines were found to be available at the district offices and at a few health facilities, but many informants complained of not having received a copy of the updated recommendations;

“*The national staff may change the guidelines but we implementers never get them. You see? Recently we were told that there is a preventive package outlined in the national strategic plan of 2009. We implementers have never seen it. But there [at national and regional level]. . . they have it*” (IDI-district informant).

This problem was also reported by the health facility staff: “*There is a problem in the distribution of the guidelines. Some of them are not available at our health facilities*” (IDI-health facility informant)*.* The same complaints emerged in the group discussion with health workers at faith-based health facility who described receiving little attention from the district in terms of getting copies of the continuously changing guidelines, giving them no chance of retrieving updated information.

## Discussion

In the discussion of the study findings, we will draw upon Rogers well-established ‘diffusion of innovation theory’ [13] as it seems particularly suited to highlighting key challenges that emerged in the study. Use of FGDs and IDIs have helped to elicit views from different informants related to the communication of infant feeding guidelines, the views that can be well linked with Rogers theory. Sanson-Fisher [36] argues that Rogers’ ‘diffusion model’ is particularly useful in providing insights into why some practices change as a response to particular health interventions while others do not. We shall attempt to assess the PMTCT guidelines as revealed in our findings in terms of (1) ‘relative advantage’, (2) ‘compatibility’, (3) ‘complexity’, (4) ‘trialability’, and (5) ‘observability’, to recall the theory’s five elements. First, we will briefly consider the meaningfulness of each of these aspects in the context of our research findings.

### Relative advantage

‘Relative advantage’ turns our attention to the degree to which ‘an innovation is perceived as better than the idea it supersedes’ ([13] p. 212), and as such indicates the benefits and costs resulting from the adoption of the innovation. This point emerges as immediately relevant when assessing the serious confusions and misunderstandings documented in this and other studies pertaining to the implementation of the many changing WHO infant feeding recommendations [37]. The proposed changes were, as we have seen above, not always perceived as better than the practice they were to replace. The recommendation that an HIV-infected mother should not breastfeed her baby if replacement feeding was ‘acceptable, feasible, affordable, sustainable and safe’ (AFASS), was clearly perceived as a problematic given that many mothers in low income contexts cannot afford infant formula [38,39]. The information that HIV-infected mothers - for whom replacement feeding was ‘not AFASS’ – should exclusively breastfeed, knowing that there is HIV in breast milk, was similarly perceived as a problematic and frightening idea.

This indicates that there was doubtful side to each infant feeding recommendation.

It is important to recall that this particular PMTCT policy (2000–01) was introduced in a context of devastating experiences of AIDS-related death in large parts of sub-Saharan Africa, and was combined with increasing awareness that HIV-infected mothers in rich parts of the world were counselled not to breastfeed in order to avoid HIV-transmission to their infants. In a context where the horrors of mass AIDS-related death in infants was feared, it was the message of replacement feeding as the only option that was preached. The confusion regarding the policy of early and abrupt cessation of breastfeeding, and the later policy preaching against giving infants animal milk produced similar confusion.

Although the response on the increased risk of HIV infection due to abrupt cessation was interpreted as misunderstanding, the later study that was published 2013 has revealed a similar scenario whereby HIV concentration increased in breast milk after breastfeeding cessation than when breastfeeding was continued [40], revealing the validity of the quote provided in the Results section. The confusion can again be explained by the fact that the recommendations could hardly be perceived as having a ‘relative advantage’ compared with those they were to replace. Relative advantage is seen by Rogers as a prime indicator of success.

### Compatibility

The second element in Rogers’ theory – ‘compatibility’ – emerges in our material as closely related to ‘relative advantage’, and refers to the degree to which an innovation is seen as compatible with the existing values, past experiences, and needs of the target population ([13] p. 224). This relates to the fundamental recognition that decision-makers have to consider the relevance and appropriateness of an intervention for any local setting. Let us briefly recall a few examples from the study to discuss the relevance of this point. Although regional managers perceived earlier on that avoidance of breastfeeding was the only solution in preventing HIV infections in infants, there was a very quick realization that dependence on infant formula was incompatible with the lives of the HIV-infected mothers, for a complex mix of economic, social and cultural reasons. In short, formula milk was too expensive. In addition, the PMTCT programme was confronted with the normative position of breastfeeding, and its deep-seated connection to motherhood within which the practice is embedded [31,39,41,42].

Beyond the lack of congruence between the various policy recommendations and the local cultural and economic context, the study findings suggest a lack of compatibility between the policy changes’ massive demands for communication and the realities on the ground. The frequent and radical changes proposed in the guidelines had to reach and be understood not only by key individuals in the PMTCT chain, but also by a vast network of stakeholders within and beyond the health system. The study findings reveal that the means and structures needed to facilitate such complex communication of information were not in place.

At one level the challenge was related to the lack of sufficient training. The workshops that were organized to educate PMTCT managers and care providers were characterized by top-down teaching; care providers were given little opportunity to question and comment upon the feasibility of the interventions. Rogers’ theory emphasizes that one-way communication - from ‘experts’ to stakeholders – is problematic because it leads to information being transferred from the ‘source’ to the receiver without discussion ([13] p. xvi).

In other studies conducted in Africa, health workers have similarly reported limited capacity to carry out proper infant feeding counselling due to lack of supportive supervision and inadequate knowledge and skills [43,44]. Without a general understanding of the rationale behind the continual changes, health managers and healthcare providers felt uncertain and found it difficult to implement the new policies. The demands for continuous updating and training implied in the PMTCT programme were simply not matched with the resources and structures encountered on the ground. The lack of compatibility between the many proposed changes in infant feeding and locally established values and practices, as well as the inability of existing structures to ensure dissemination of the information created a most challenging backdrop for the introduction of new recommendations.

### Complexity

Rogers’ ‘complexity’ concept refers to ‘the degree to which an innovation is perceived as difficult to understand and use’ ([13] p. 242). He suggests that clinical procedures are ‘more likely to be adopted if they are simple and well defined.’ With reference to our material; the scientific knowledge underpinning the many and diverse PMTCT policies has simply not been easy to understand. It is, for example, challenging to explain that ‘there is HIV in your breast milk which can infect your infant, but if you *only* breastfeed the likelihood of transmission is very low’. It was also highly challenging for the health workers to relate to the demanding assessment implied by the AFASS criteria. The many and radical policy shifts seriously complicated the process of presenting key concepts in a straightforward manner.

The sense of complexity in the intervention has been increased by the lack of simple information and instruction materials. The study informants referred to the large and complex PMTCT manuals written in language that was not easily comprehensible to people without higher education. The manuals were written in English rather than in Swahili, the *lingua franca* of Tanzania, and the language barrier was naturally seen to add to the knowledge gap. Proper translation of the recommendations was therefore not secured. Neither did the informants find any guides or pamphlets that communicated key policy information including the evidence or reasons behind the policy changes. These findings are contrary to what Jackson and Feder recommend as vital, namely that the evidence and recommendations should be presented in a concise, accessible manner and in a format that makes it possible for the implementers to understand and enforce them [3]. Jackson and Feder’s ideas are in line with what Rogers advocates under the ‘complexity’ element. Our finding that there is a lack of a “reading culture” among health workers emphasises the importance of simple and clear information. Bowen [45] in his study from Canada points out how the language barrier contributes to low participation of stakeholders in various interventions, increases the risk of misdiagnosis, poor patient understanding of the recommendations, low patient satisfaction, and low quality of care with implications for poor health outcomes. Brinkher and Crosby (2002) argue that the development of a strong rapport requires the use of a language that makes the proposed recommendation understandable and appealing to potential supporters [46]. Others have likewise suggested that a main requirement of a successful implementation process is producing guidelines in a user-friendly format [3,47].

Rogers’ diffusion of innovation theory stipulates that complexity in communicating new policy may arise from the complexity of the innovation itself or from its modality of delivery. Findings from our study indicate that, in the PMTCT programme, both the policy itself and the delivery mode have been challenging, and have matched poorly with the realities on the ground. This has led to confusion and frustration among health managers, care providers and HIV infected mothers alike.

### Trialability

Trialability refers to the degree to which the proposed innovation is tested to assess how it works in the specific conditions ([13] p. 243). Trialability thus aims at exploring the feasibility of the implementation of the procedure, its acceptability to patients, and the potential outcomes prior to its intervention. The frequent policy changes in the WHO’s infant feeding recommendations in the PMTCT programme point to a lack of sufficient evidence relating to the outcomes of the intervention. Before the PMTCT programme was scaled up in Tanzania, it was piloted in a few referral hospitals, but the last decade has clearly proven that the pilot was not sufficient to avoid massive confusion and frustration during the implementation of the programme. The review by Van de Perre et al. [10] argues that, in the case of the PMTCT programme, the WHO produced global recommendations which lacked the necessary scientific basis in terms of clinical trials, and was largely based upon ‘expert’ opinion.

### Observability

Rogers writes that the observability of positive outcomes of an innovation is positively related to its rate of adoption ([13] p. 244). The question of observation of positive or productive outcomes of the many infant feeding recommendations was not discussed in a direct manner with the study participants. However, the observable negative outcomes of the policy of replacement feeding as first choice have been reported by Moland and Blystad [39], and shows that hardships produced by the PMTCT policy led to continuous attempts at modification. Informants, in our study, would mention the trust they had developed in recent years towards the recommendation of exclusive breastfeeding, pointing at the observable outcomes of HIV free babies who were born and breastfed by HIV infected mothers. This immensely important observation has been vital in a PMTCT context. The success of the recent emphasis on extended breastfeeding and complementary feeding has, in a powerful manner, demonstrated the significance of the Rogers’ other points: the breastfeeding policy was seen to have a relative advantage over earlier recommendations; it was compatible with local culture; it did not imply a complex message; and it has been tried out as a successful strategy for infant feeding; for thousands of years.

#### Study limitations and strengths

This study has several limitations. The sample size is limited as it was carried out merely within one district in Tanzania and it did not include the national level which could have informed the study of PMTCT policy dissemination and communication in important ways. The beneficiaries (the women) were not included in this study because the aim was to focus to health workers and managers who were to communicate the messages of the frequent changes of the guidelines to the lower levels. Despite these limitations, we believe that the study findings may be of interest and relevance for other Tanzanian districts dealing with the communication of PMTCT related policy. We suggest that the findings may potentially be of interest to the study of communication of health related policy or guidelines at a more general level, particularly within weak health systems.

## Conclusion

The study has revealed substantial challenges in the implementation of changing global HIV and infant feeding guidelines among healthcare managers at regional, district and health-facility level. The challenges were related to the incompatibility of the infant feeding recommendations with the local socio-economic context but also to factors beyond that. Structural barriers generated severe challenges to the dissemination of information within the programme. Current HIV and infant feeding guidelines have turned in a direction that is more compatible with local perceptions and practices, and it is more feasible for the health system and the HIV-infected mothers to relate to them. This generates hopes that lessons have been learnt in terms of the multiple challenges that are likely to arise in when new and updated clinical guidelines are introduced at ever-increasing speed within weak health systems.
